# Country-scale diversity, spatial genetic structure, and mitochondrial demographic history of *Aedes aegypti* populations in Colombia

**DOI:** 10.1371/journal.pntd.0014808

**Published:** 2026-10-13

**Authors:** Andrés Gómez-Palacio, Johana Marín-Suarez, Laura Beltrán-Rodríguez, Marcela Gómez, Weimar Briñez-Zabaleta, Abelgie Sánchez, Luz Helena Patiño, Sandra Uribe-Soto, Juan David Ramírez

**Affiliations:** 1 Laboratorio de Investigación en Genética Evolutiva (LIGE), Universidad Pedagógica y Tecnológica de Colombia, Tunja, Boyacá, Colombia; 2 Grupo de Estudios en Genética y Biología Molecular (GEBIMOL), Universidad Pedagógica y Tecnológica de Colombia, Tunja, Boyacá, Colombia; 3 Centro de Investigaciones en Microbiología y Biotecnología-UR (CIMBIUR), School of Sciences and Engineering, Universidad del Rosario, Bogotá, Colombia; 4 Grupo de Investigación en Ciencias Básicas (NÚCLEO), Facultad de Ciencias e Ingeniería, Universidad de Boyacá, Tunja, Colombia; 5 Grupo de Investigación en Sistemática Molecular (GSM), Universidad Nacional de Colombia, Medellín, Colombia; 6 Center for Global Health and Inter-Disciplinary Research, USF Genomics Program, Department of Global, Environmental and Genomic Health Sciences, College of Public Health, University of South Florida, Tampa, Florida, United States of America; Gulu University Faculty of Science, UGANDA

## Abstract

*Aedes aegypti* is the primary urban vector of major human arboviruses worldwide, yet the spatial genetic structure and demographic history of Colombian populations remain incompletely understood. Here, we analyzed mitochondrial *cytochrome c oxidase* subunit I (COX1) sequences from Colombian populations sampled across a broad geographic and temporal range to evaluate genetic diversity, haplotype distribution, spatial-temporal structure, and mitochondrial demographic history. The dataset combined publicly available COX1 records with 394 newly generated sequences from Colombian specimens, yielding 717 entries from the Caribbean, Andean, Orinoco, and Amazonian regions of Colombia, and was analyzed using population genetic, ordination, constrained spatial, and demographic inference approaches. Colombian COX1 diversity was mainly structured into two broad mitochondrial genetic clusters rather than by sampling locality, department, or collection year, suggesting the persistence of maternal lineages together with spatial mixing likely influenced by human-mediated dispersal and urban adaptation. Human population density and built-up intensity partially explained mitochondrial genetic structure, but this association varied through time, indicating that urbanization interacts with regional demographic heterogeneity and local habitat configuration. Demographic history analyses suggested temporal variation in mitochondrial effective population size, including an older increase followed by reduction and relative stabilization toward recent generations, rather than continuous demographic expansion. Climate-associated analyses further suggested that temperature, particularly minimum temperature, may contribute to mitochondrial demographic variation, although this relationship should be interpreted as correlative. Overall, Colombian *Ae. aegypti* populations appear to be shaped by historical maternal-lineage structure, urbanization-associated spatial heterogeneity, eco-regional demographic variation, and climatic context. This country-scale mitochondrial baseline can help prioritize regions for genomic, entomological, and epidemiological surveillance and guide future investigations of mosquito dispersal and population connectivity relevant to vector management in Colombia.

## Author summary

*Ae. aegypti* is the main urban mosquito vector of dengue and other arboviruses, but its population history in Colombia remains incompletely understood. We analyzed 717 COX1 mitochondrial DNA sequences from mosquitoes collected or reported across the Caribbean, Andean, Orinoco, and Amazonian regions to examine how genetic diversity is distributed across the country. Colombian populations were mainly organized into two broad mitochondrial clusters, suggesting that historical maternal lineages persist but are widely mixed through human movement, urban connectivity, and repeated colonization of domestic habitats. Urbanization and built-up intensity explained part of this genetic structure, although their effects varied over time and among regions. Demographic analyses also suggested temporal changes in mitochondrial effective population size rather than continuous expansion, and temperature, especially minimum temperature, may contribute to this variation. These findings are relevant in light of recent INS dengue surveillance, which identified regionally distinct dengue-incidence clusters, with Amazonian, Caribbean, Orinoco, and Andean municipalities following different epidemic trajectories. Overall, our results suggest that Colombian *Ae. aegypti* populations are shaped by historical lineage structure, urbanization, regional ecology, climatic context, and epidemiological heterogeneity, providing a mitochondrial baseline for future genomic and vector-surveillance efforts.

## Introduction

*Aedes aegypti* is the primary urban vector of major human arboviruses worldwide, including dengue, Zika, chikungunya, and yellow fever, and it remains a major public health concern in Colombia [1]. Recent genomic evidence has reinforced the view that *Ae. aegypti* has a complex global history of origin, movement, and epidemiological expansion, emphasizing the importance of reconstructing its dispersal and demographic patterns across endemic regions [2]. Likewise, genomic analyses suggest that present-day New World populations of *Ae. aegypti* derive from a human-specialist lineage that diverged in Africa approximately 5,000 years ago and later spread to the Americas from West Africa during the Atlantic Slave Trade [3].

In Colombia, this relevance is reflected by the continued public health importance of dengue and its recurrent transmission across multiple regions of the country [4]. In this context, population genetic approaches provide a useful framework for reconstructing historical processes from present-day patterns of genetic variation, particularly when these are examined in a geographic setting. Analyses of haplotype distribution, lineage relationships, and population structure can therefore contribute to understanding dispersal history, spatial differentiation, and demographic change in vector species [5–8]. From an epidemiological perspective, understanding demographic history may help explain how *Ae. aegypti* populations persist through time, spread across regions, and re-establish after control efforts, all of which are relevant to the dynamics of arbovirus transmission in Colombia.

Among the molecular markers used in insect population studies, mitochondrial DNA has been particularly informative for assessing phylogeographic structure and demographic history. Because mitochondrial haplotypes can retain signatures of lineage diversification and population size changes, markers such as *cytochrome c oxidase* subunit I (COX1) remain useful for large-scale evolutionary analyses, especially when newly generated data are integrated with historical records available in public repositories [9,10]. Gene genealogies, haplotype frequencies, and their spatial distribution have long been used to infer demographic events such as expansion, fragmentation, and range shifts [11–16].

In Colombia, previous studies have documented genetic differentiation among populations from northern municipalities with contrasting dengue incidence, as well as broader patterns of lineage turnover, ancestry, and genomic structure, supporting a historically dynamic view of national populations [17–20]. Previous studies have shown that Colombian populations of *Ae. aegypti* harbor a complex genetic background. Mitochondrial analyses revealed spatio-temporal variation in lineage composition among Colombian cities with distinct dengue incidence patterns, suggesting complex population dynamics and heterogeneous lineage persistence [21]. Subsequent ancestry-based analyses supported a single African origin of Colombian populations, emphasizing the relevance of historical founding processes in shaping present-day structure [22]. More recently, comparisons between SNP-chip and whole-genome sequencing datasets showed that population-structure and demographic inferences in *Ae. aegypti* are robust across marker systems [19]. COX1 was selected because it is a standardized mitochondrial marker with extensive representation in public databases, allowing the direct integration of newly generated sequences with geographically and temporally dispersed Colombian records. Its maternal inheritance, limited recombination, and combination of conserved and variable sites make it informative for evaluating haplotype diversity, maternal-lineage distributions, and historical phylogeographic patterns [23–25]. However, as a single mitochondrial locus, COX1 does not capture genome-wide population structure, contemporary biparental gene flow, or nuclear admixture. Accordingly, this study provides an updated country-scale synthesis of mitochondrial COX1 variation in Colombian *Ae. aegypti*, rather than a genome-wide assessment of population structure.

Additional records from regions such as Arauca further show that Colombian molecular data have accumulated under diverse surveillance and identification contexts, but remain fragmented from a country-scale phylogeographic perspective [20]. At the same time, mitochondrial information for *Ae. aegypti* has accumulated from geographically scattered studies across Colombia. Molecular surveys and sequence-based identifications have documented specimens from departments such as Arauca, Antioquia, and Vichada, among others, under different research aims including arbovirus surveillance, DNA barcoding, and local vector characterization [20,26–34]. Although valuable, these records remain fragmented from a national phylogeographic perspective. Their integration into a unified dataset provides an opportunity to reassess mitochondrial diversity across Colombia using broader geographic coverage and a common analytical framework.

In this study, we combined publicly available COX1 sequences with newly generated sequences from Colombian *Ae. aegypti* specimens to evaluate genetic diversity, haplotype distribution, and demographic history at a country scale. Specifically, we examined the spatial distribution of mitochondrial haplotypes and applied population genetic and demographic inference approaches to test whether Colombian populations show signatures of historical expansion, stability, or fluctuation in effective population size. By integrating broad geographic sampling with historical sequence information, this study provides an updated phylogeographic perspective on *Ae. aegypti* in Colombia and contributes to a better understanding of the evolutionary background of one of the world’s most important arboviral vectors.

## Materials and methods

### Ethical considerations

This study was conducted using mosquito specimens and publicly available sequence data. No human participants, human biological samples, clinical records, or personally identifiable information were used. Newly generated *Aedes aegypti* sequences were obtained from mosquito specimens collected as part of entomological surveillance and/or research activities, and the analyses were restricted to vector genetic data. Therefore, the study did not require approval from a human-subjects ethics committee. When applicable, mosquito collection and use of biological material followed institutional procedures and national regulations for scientific research on biological diversity in Colombia. Publicly available COX1 sequences were retrieved from GenBank and analyzed together with newly generated data, with original accession information retained for traceability.

### Insect samples and data collection

A total of 717 COX1 records of *Ae. aegypti* from 25 municipalities distributed across 12 departments of Colombia and collected between 2012 and 2024 were considered in this study (Table 1 and S1 Fig). Of these, 394 corresponded to newly generated sequences obtained from specimens collected in urban localities, whereas 317 were publicly available records retrieved from GenBank. For the newly generated dataset, adult mosquitoes were collected using entomological nets and BG-Sentinel traps (Biogents AG, Regensburg, Germany), depending on local field conditions. Specimens were preserved and transported under cold-chain conditions following previously described procedures [18,21], and morphologically identified using a standard pictorial taxonomic key [35]. Confirmed *Ae. aegypti* specimens were subsequently processed individually for molecular analysis.

**Table 1:** Summary of the 717 *Aedes aegypti* COX1 sequences analyzed across four Colombian eco-regions, including locality, sampling period, data source, accession status, and geographic coordinates.

Eco-region	Department	Locality	No. sequences	Collection years	Sequence source	Latitude	Longitude	Number in map
Amazon	Amazonas	Leticia	78	2021	This study	−4.2211	−69.9431	1
Subtotal			78					
Andean	Antioquia	Bello	57	2012-2013	GenBank	6.3333	−75.5833	2
		La Pintada	5	2014	GenBank	5.7401	−75.6001	3
		Medellín	14	2015-2019	GenBank/ This study	6.2518	−75.5636	4
		Puerto Triunfo	20	2024	This study	5.8991	−74.7352	5
	Boyacá	Moniquirá	20	2023	This study	5.8764	−73.5728	6
		Muzo	20	2023	This study	5.5353	−74.1078	7
		Otanche	45	2020-2023	This study	5.6567	−74.1825	8
		Puerto Boyacá	34	2020-2023	This study	5.9758	−74.5885	9
	Quindío	Armenia	17	2014-2015	GenBank	4.5201	−75.7001	10
		Circasía	10	2014	GenBank	4.6101	−75.6101	11
	Tolima	San Luis	78	2024	This study	4.1334	−75.0924	12
Subtotal			320					
Caribbean	Bolívar	Cartagena	27	2022	This study	10.3922	−75.4921	13
	Córdoba	San Bernardo del Viento	7	2013	GenBank	9.3501	−75.9401	14
	La Guajira	Riohacha	58	2012-2013	GenBank	11.5167	−72.9167	15
	Magdalena	El Retén	29	2021	This study	10.6111	−74.2691	16
		Santa Marta	5	2018	GenBank	11.2396	−73.7995	17
Subtotal			126					
Orinoco	Arauca	Arauquita	9	2018-2019	GenBank	7.0845	−70.7554	18
		Saravena	59	2018-2019	GenBank	6.9632	−71.8823	19
		Tame	23	2018-2019	GenBank	6.4606	−71.7362	20
	Meta	Villavicencio	9	2012-2021	GenBank/ This study	4.0667	−73.6667	21
	Vichada	Cumaribo	14	2016	GenBank	4.4455	−69.799	22
		La Primavera	23	2016	GenBank	5.4906	−70.4092	23
		Puerto Carreño	16	2021	This study	6.19	−67.4782	24
		Santa Rosalia	40	2016-2021	GenBank/ This study	5.1269	−70.8771	25
Subtotal			193					
Total			717					

After global alignment, six GenBank accessions (PV034759.1, MN299011.1, MN299013.1, PV544864.1, PV544865.1, and PV544866.1) were excluded because they were highly divergent from the remaining dataset, suggesting possible quality or identification errors. Thus, 311 GenBank sequences were retained. An additional 12 metadata records were then generated from shared haplotypes reported by Jaimes-Dueñez et al. (2015), corresponding to locality-year combinations in Bello (Antioquia), Riohacha (La Guajira), and Villavicencio (Meta), resulting in 323 GenBank-derived records in the curated dataset. Together with the 394 newly generated sequences, these records yielded a final dataset of 717 entries spanning the Caribbean (departments of Bolívar, Córdoba, La Guajira, and Magdalena), Andean (Antioquia, Boyacá, Quindío, and Tolima), Orinoco (Arauca, Meta, and Vichada), and Amazonian (Amazonas) regions of Colombia. Associated metadata included department, municipality, latitude, longitude, collection year, original sample code, GenBank accession number when available, and source of origin. The complete accession-level metadata are presented in S1 Table.

Sequence identifiers were standardized to include the department and locality of origin, followed by a consecutive number (e.g., VicCum_01 for specimen 1 from Cumaribo, Vichada). Metadata were retrieved from GenBank in R using the *rentrez* package, and records were processed in batches to comply with NCBI request limits (S1 Data). When accession-matched external metadata were available, department, locality, geographic coordinates, collection year, and original sample codes were completed from the curated dataset assembled for this study. For accessions associated with shared haplotypes across multiple sampling events, particularly those reported by Jaimes-Dueñez et al. (2015), the same haplotype was assigned to each locality-year combination in the metadata table to preserve consistency in haplotype inference while retaining the geographic and temporal representation of the dataset.

### DNA extraction, PCR amplification, and sequencing

For the 394 sequences newly generated in this study, mosquito tissues were processed for molecular analyses following general procedures previously used in Colombian *Aedes aegypti* studies for mitochondrial marker characterization and arbovirus-related screening [18,21]. DNA was extracted from whole individual mosquitoes. Accordingly, extracts from inseminated females could theoretically contain trace amounts of male-derived DNA from stored sperm. However, because female tissues constitute the predominant source of mitochondrial DNA, this contribution was considered unlikely to materially affect COX1 consensus sequences or population-level haplotype analyses. Because some specimens were also included in complementary screening workflows, the extraction procedures were designed to yield material suitable for downstream PCR-based applications [18]. For the purposes of this study, only the amplification and sequencing of the mitochondrial COX1 marker are described below.

A fragment of the mitochondrial cytochrome c oxidase subunit I (COX1) gene was amplified using the universal barcoding primers LCO1490 (5′-GGTCAACAAATCATAAAGATATTGG-3′) and HCO2198 (5′-TAAACTTCAGGGTGACCAAAAAATCA-3′) [36]. PCR amplification followed a common thermal profile across samples, consisting of an initial step at 50 °C for 10 min, followed by denaturation at 95 °C for 1 min, 45 cycles of 94 °C for 10 s and 60 °C for 1 min, and a final extension at 72 °C for 10 min. Amplification products were evaluated on 2.5% agarose gels in 1 × TBE buffer stained with SYBR Safe (Invitrogen, Carlsbad, CA, USA). Samples showing the expected fragment of approximately 650 bp were selected for Sanger sequencing.

### Data processing

Sequence processing and quality control were performed iteratively during assembly of the final dataset. Sequences were first cleaned by removing gap characters prior to similarity screening. The resulting ungapped sequences were then validated against the *Ae. aegypti* mitochondrial COX1 reference region (NC_035159.1:1298–2834), corresponding to the mitochondrial annotation associated with the AaegL5.0 reference assembly [37], using BLASTN. For each retained record, only the BLAST-supported query regions were preserved, and the filtered sequences were exported as a new FASTA file. These sequences were subsequently aligned using MAFFT v7.525 [38].

Because the dataset comprised partial COX1 fragments generated from different studies and primer combinations, variation in sequence length was expected mainly at the alignment ends rather than across the internal homologous region. Gap-threshold exploration was therefore used primarily to identify and trim poorly aligned terminal regions while preserving the longest informative shared segment of the alignment. Gap thresholds from 10% to 90% were evaluated in 5% increments by comparing retained alignment positions and mean ungapped sequence length. Because all 717 sequences were retained across thresholds, the final selection was based mainly on the balance between alignment length and gap removal. Based on this trade-off analysis, a threshold of 45% was selected, retaining 591 positions and a mean ungapped sequence length of 532.16 bp (S2A Fig). Alignment positions with gaps in 45% or more of the sequences were removed, yielding the final filtered alignment used in downstream analyses. Most highly gapped sites were concentrated at the alignment ends, with fewer isolated internal positions (S2B Fig). The distribution of ungapped sequence lengths across the dataset is shown in S2C Fig. Diagnostic plots summarizing threshold performance, critical gap positions, and sequence-length distribution were generated as part of this workflow (S2 Data).

As an additional quality-control step, the final alignment was checked for coding-frame integrity using a custom R script. After residual gap characters and non-ACGT symbols were removed, sequences were translated under the invertebrate mitochondrial genetic code. Internal stop codons were identified and, where possible, corrected using consensus-based codon replacement. The repaired sequences were then retranslated to confirm the absence of internal stop codons, and the corresponding protein translations were further validated using BLASTP (S3 Data).

### Population genetic diversity and genetic structure analyses

Population genetic diversity was estimated from the final curated COX1 alignment by assigning the 717 *Ae. aegypti* sequences to their corresponding department, locality, and geographic coordinates. Locality-level haplotype diversity (Hd), nucleotide diversity (π), and Watterson’s theta (θW) were estimated in R using the *pegas* [39] and *ape* [40] packages. Spatial patterns of genetic diversity were visualized by linking diversity estimates to locality coordinates using *sf* [41,42] and *ggplot2* [43], and exploratory interpolated surfaces were generated with the *akima* package [44]. A median-joining haplotype network was constructed to examine relationships among COX1 haplotypes [45]. Before network reconstruction, star contraction was applied with a radius of 5 to reduce network complexity by collapsing closely related haplotypes [46]. To further resolve reticulations and simplify the network, maximum-parsimony post-processing was applied after the median-joining procedure [47]. The network was generated in Network v.10 (https://www.fluxus-engineering.com/sharenet.htm) using default parameters.

Genetic structure among individuals was evaluated using pairwise Kimura two-parameter (K2P) distances [48]. To explore mitochondrial genetic structure, we used a data-driven clustering approach based on pairwise Kimura 2-parameter distances among individual COX1 sequences. For this clustering analysis, no groups were predefined according to locality, department, geographic region, collection year, or previously reported mitochondrial lineage. This approach avoided imposing discrete population boundaries, given previous microsatellite evidence of substantial gene flow and limited nuclear differentiation among geographically separated Colombian populations [22].

The K2P matrix was transformed by classical multidimensional scaling, and the first two MDS axes were analyzed using k-means clustering. The optimal number of clusters was assessed using an elbow analysis based on the total within-cluster sum of squares across an exploratory range of k values from 1 to 30. Cluster assignments were exported for downstream visualization and interpretation.

### Spatial genetic structure

Spatial genetic structure was assessed using the individual-level K2P genetic-distance matrix derived from the final COX1 alignment. Non-metric multidimensional scaling (NMDS) was performed with the *vegan* package [49] to visualize genetic dissimilarities among individuals and to explore their association with department, locality, collection year, and inferred genetic clusters. The contribution of spatial and temporal variables to genetic structure was evaluated using PERMANOVA and variation partitioning in *vegan* [49]. Administrative variables, including department and locality, were used as explanatory or annotation factors, whereas clustering analyses were based on genetic distances and did not impose predefined geographic groups.

Hierarchical genetic structure was further assessed using AMOVA implemented in the *pegas* package [39]. The hierarchy comprised the two K2P-derived mitochondrial clusters as the upper level, departments nested within clusters, and collection years nested within departments. Thus, the analysis partitioned variance among clusters, among departments within clusters, among years within departments, and within the remaining individual-level variation. Isolation by distance was evaluated by comparing pairwise K2P genetic distances with pairwise geographic distances calculated from latitude and longitude coordinates using the *geosphere* package [50]. In addition, distance-based redundancy analysis (dbRDA) was performed with *vegan* [49] to examine the joint contribution of spatial coordinates and collection year to genetic-distance variation.

### Demographic history inference and ecological-climatic associations with inferred effective population size

Historical changes in the effective population size (Ne) of *Ae. aegypti* in Colombia were inferred from the final curated COX1 alignment using a coalescent-based Bayesian Skyline Plot (BSP) implemented in BEAST2 [51]. Analyses were run through the CIPRES Science Gateway using the BEAST2 implementation available on the platform [52]. The analysis was performed under a strict molecular clock and the HKY + G nucleotide substitution model [53]. Temporal scaling used the conventional arthropod mitochondrial COI rate of 2.3% pairwise divergence per million years, equivalent to 0.0115 substitutions per site per million years per lineage [54,55]. Because mitochondrial evolutionary rates vary among insect lineages and calibration frameworks [56], the resulting dates were interpreted as approximate and calibration-dependent estimates rather than precise chronological events. Markov chain Monte Carlo analyses were run for 100 million generations, with parameters sampled every 10,000 generations. Convergence and mixing were assessed in Tracer v1.7.2 [57] by verifying effective sample size values for the relevant parameters. Skyline outputs from independent runs were combined and summarized in R using *tidyverse* [58], extracting posterior estimates of Ne and their 95% highest posterior density intervals. The BSP was then visualized to infer historical changes in Ne through time.

To explore whether temporal variation in BSP-inferred Ne was associated with broad ecological and climatic conditions, we compared Ne estimates with environmental descriptors across Colombia. Climatic variables were obtained from WorldClim layers for 2000–2024, including precipitation, minimum temperature, and maximum temperature [59]. Raster values were extracted at 2.5 arc-min resolution, corresponding to approximately 21 km^2^, and summarized as annual and monthly climatic estimates. Mean annual Ne values were compared with mean annual climatic variables, whereas interpolated Ne estimates were compared with monthly climatic values to evaluate finer temporal associations. Ecological context was assessed using Colombian biome/ecoregion categories based on IDEAM and IAvH (2024; http://www.ideam.gov.co/web/ecosistemas) spatial layers. Sampling localities were assigned to their corresponding ecological categories by spatial intersection. Associations between Ne and climatic variables were assessed using Spearman rank correlations across the full dataset and after stratification by Colombian ecoregions. These analyses were considered exploratory because some ecological categories were represented by few observations. All analyses and visualizations were performed in R (S4 Data).

### Anthropogenic landscape association with genetic structure

To evaluate whether human population density was associated with mitochondrial genetic structure, population data were extracted from Global Human Settlement Layer (GHSL) population raster products for 2010, 2015, and 2020. Raster tiles were downloaded from the Copernicus Global Human Settlement Layer repository (https://human-settlement.emergency.copernicus.eu/), mosaicked, cropped to the Colombian study area, and reprojected to the Mollweide coordinate reference system. Sampling coordinates were transformed to the same projection, and raster values were extracted at each sampling point using the *terra* [60] and *sf* [41,42] packages in R. Extracted values were converted to estimated population density expressed as people per km^2^.

Population-density values were classified into four categories: sparse (<500 people/km^2^), semi-urban (500–1,999 people/km^2^), urban (2,000–9,999 people/km^2^), and highly urban (≥10,000 people/km^2^). Sampling points without available raster information were retained as “No data” when applicable. These categories were used to visualize the distribution of samples across different levels of human population density.

The association between genetic structure and human population density was evaluated using dbRDA in the *vegan* package [49]. The individual-level K2P genetic-distance matrix was used as the response variable, and population density was used as the explanatory variable. Models were fitted separately by collection year and for the complete dataset. In addition, PERMANOVA was performed to test whether genetic distances differed among population-density classes using *vegan* package [49]. Ordination plots and model summaries were generated with *ggplot2* [43].

## Results

### Genetic variation and diversity

All genetic diversity estimates are summarized in Table 2. Across the complete COX1 dataset, the 717 *Ae. aegypti* sequences contained 60 segregating sites and 81 haplotypes, with moderate haplotype diversity (Hd = 0.589 ± 0.02), nucleotide diversity (π = 0.0176 ± 0.001) and Watterson’s theta (θW = 82.7), indicating substantial mitochondrial polymorphism at the country scale. Haplotype diversity (Hd) varied markedly among the 25 sampled localities, ranging from 0.000 to 0.993 (Fig 1a, 1b). The highest Hd values were observed in El Retén, Puerto Carreño, Otanche, Bello, and San Luis, whereas Circasía and Cumaribo showed no haplotype diversity. Nucleotide diversity (π) ranged from 0.000 to 0.0778 (Fig 1c, 1d), with the highest values detected in Cartagena, Otanche, San Luis, La Pintada, and Saravena, and no nucleotide diversity detected in Circasía and Cumaribo. Watterson’s theta (θW) ranged from 0.00 to 147.4 (Fig 1e,1f), with the highest estimates observed in Santa Marta, Cartagena, Otanche, Santa Rosalía, and Moniquirá, while Circasía and Cumaribo again showed θW = 0.00. Overall, Hd, π, and θW showed spatially heterogeneous distributions across Colombia, with localized peaks of mitochondrial diversity and segregating-site variation rather than a continuous north–south or east–west geographic gradient.

**Table 2:** Locality-level COX1 diversity and haplotype composition of Colombian *Aedes aegypti*. Haplotype diversity (Hd), nucleotide diversity (π), Watterson’s theta (θW) were calculated from the COX1 sequence alignment before haplotype-network simplification. The number and distribution of haplotypes correspond to the haplotype assignments used for network reconstruction following star contraction and maximum-parsimony post-processing. Values in parentheses indicate the number of sequences assigned to each haplotype. For departmental subtotals, SD values are reported for Hd and π only; θW is reported as a point estimate.

Dep.	Locality	No. Seqs.	Hd (SD)	π (SD)	θW	No. Haplo.	Haplotype distribution (n)
Amazonas	Leticia	78	0.87	0.016	34.3	12	H1(5); H5(61); H30(3); H31(1); H32(1); H33(1); H34(1); H35(1); H36(1); H37(1); H38(1); H39(1)
	Subtotal	78	0.87 (0.05)	0.016 (0.004)	34.3	12	
Antioquia	Bello	57	0.97	0.014	8.2	7	H1(24); H5(2); H9(27); H15(1); H17(1); H18(1); H19(1)
	La Pintada	5	0.70	0.046	32.6	2	H1(4); H12(1)
	Medellín	14	0.70	0.006	20.1	2	H1(4); H9(10)
	Puerto Triunfo	20	0.37	0.001	1.4	3	H1(17); H5(2); H20(1)
	Subtotal	96	0.92 (0.02)	0.014 (0.002)	21.2	9	
Boyacá	Moniquirá	20	0.81	0.011	107.4	3	H1(18); H28(1); H29(1)
	Muzo	20	0.83	0.031	76.9	3	H1(18); H22(1); H29(1)
	Otanche	45	0.97	0.052	125.1	17	H1(22); H20(2); H21(1); H22(1); H23(2); H24(2); H40(1); H41(1); H42(1); H43(5); H44(1); H45(1); H46(1); H47(1); H48(1); H49(1); H50(1)
	Puerto Boyacá	34	0.84	0.017	48.7	6	H1(29); H20(1); H25(1); H26(1); H27(1); H51(1)
	Subtotal	119	0.67 (0.05)	0.031 (0.004)	54.1	23	
Quindío	Armenia	17	0.23	0.001	0.6	1	H1(17)
	Circasía	10	0.00	0.00	0.0	1	H1(10)
	Subtotal	27	0.15 (0.09)	0.00 (0.000)	0.6	1	
Tolima	San Luis	78	0.96	0.049	68.8	12	H1(65); H13(1); H20(1); H23(1); H47(1); H51(3); H69(1); H70(1); H71(1); H72(1); H73(1); H74(1)
	Subtotal	78	0.96 (0.03)	0.05 (0.005)	68.8	12	
Bolívar	Cartagena	27	0.93	0.078	141.9	10	H1(6); H5(6); H9(8); H75(1); H76(1); H77(1); H78(1); H79(1); H80(1); H81(1)
	Subtotal	27	0.93 (0.04)	0.078 (0.011)	141.9	12	
Córdoba	San Bernardo del Viento	7	0.67	0.012	4.5	2	H1(4); H9(3)
	Subtotal	7	0.67 (0.16)	0.012 (0.003)	4.5	2	
La Guajira	Riohacha	58	0.94	0.005	8.6	8	H1(49); H5(3); H9(1); H13(1); H14(1); H15(1); H16(1); H17(1)
	Subtotal	58	0.94 (0.02)	0.005 (0.000)	8.6	8	
Magdalena	El Retén	29	0.99	0.033	56.0	12	H1(12); H5(1); H43(7); H46(1); H52(1); H53(1); H54(1); H55(1); H56(1); H57(1); H58(1); H59(1)
	Santa Marta	5	0.60	0.004	147.4	1	H1(5)
	Subtotal	34	0.94 (0.03)	0.037 (0.007)	101.5	12	
Arauca	Arauquita	9	0.42	0.004	3.3	1	H1(5)
	Saravena	59	0.74	0.042	13.9	4	H1(34); H5(13); H9(15); H10(1)
	Tame	23	0.52	0.002	2.2	3	H1(20); H5(2); H11(1)
	Subtotal	91	0.68 (0.05)	0.031 (0.005)	17.4	5	
Meta	Villavicencio	9	0.89	0.003	37.5	1	H1(9)
	Subtotal	9	0.89 (0.09)	0.003 (0.000)	37.5	1	
Vichada	Cumaribo	14	0.00	0.000	0.0	1	H1(14)
	La Primavera	23	0.32	0.008	10.3	4	H1(20); H6(1); H7(1); H8(1)
	Puerto Carreño	16	0.99	0.028	29.2	5	H1(10); H43(3); H66(1); H67(1); H68(1)
	Santa Rosalia	40	0.85	0.022	111.9	13	H1(25); H2(1); H3(1); H4(1); H5(1); H43(3); H59(1); H60(1); H61(1); H62(1); H63(1); H64(2); H65(1)
	Subtotal	93	0.65 (0.06)	0.018 (0.004)	35.4	19	
Total		717	0.589 (0.02)	0.0176 (0.001)	82.7	81	

**Fig 1:**
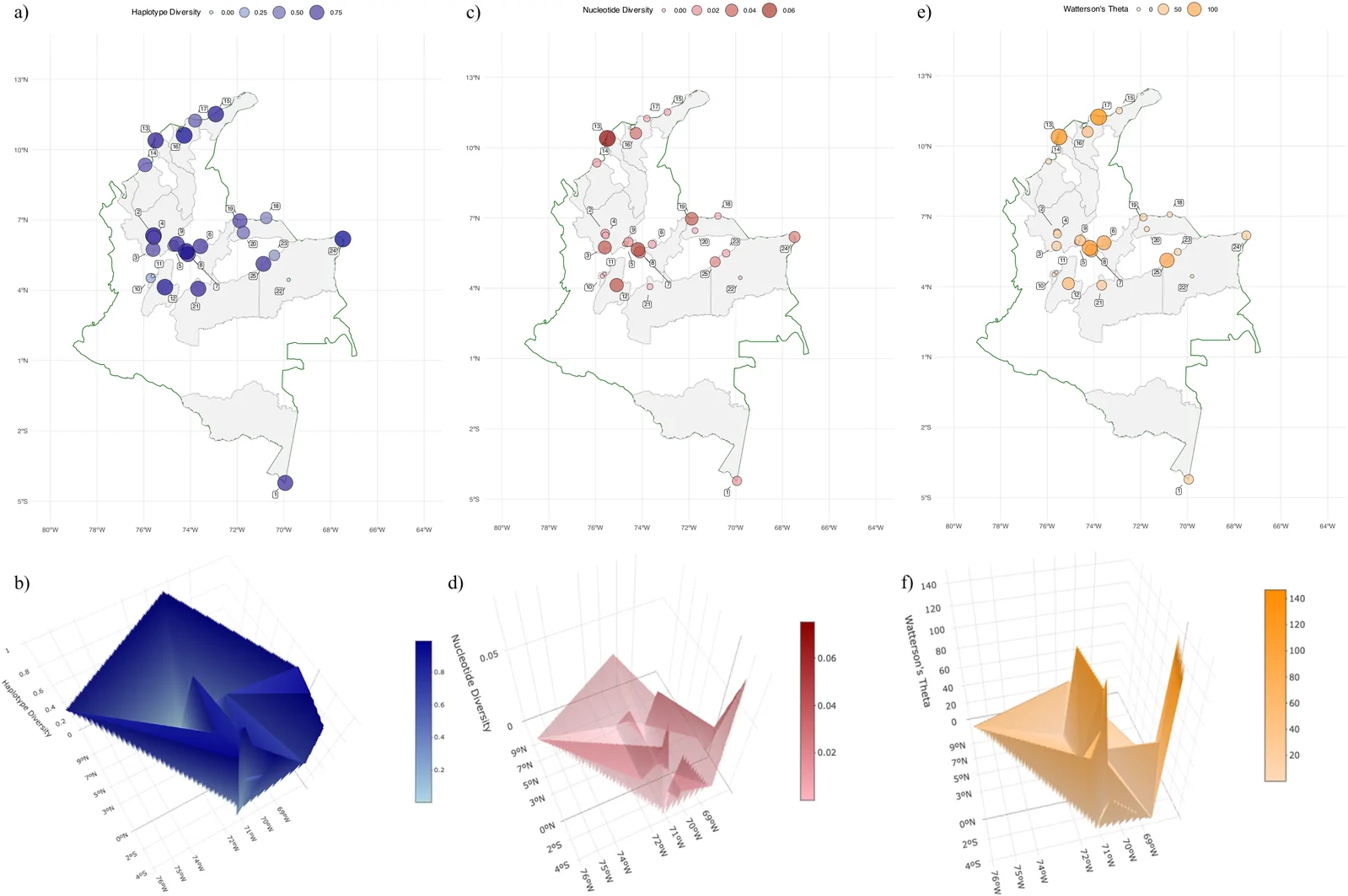
Spatial distribution of mitochondrial COX1 diversity in Colombian *Aedes aegypti* populations. Locality-level estimates are shown for haplotype diversity (Hd; a, b), nucleotide diversity (π; c, d), and Watterson’s theta (θW; e, f). In the map panels, sampled localities are identified by the numeric codes defined in Table 1, and symbol size is proportional to each diversity estimate. The corresponding interpolated surfaces illustrate the spatial distribution of each parameter across the sampled geographic range. Administrative boundaries and country outlines were obtained from Natural Earth via the `rnaturalearth` and `rnaturalearthhires` R packages. Natural Earth data are in the public domain. Data and terms: [https://www.naturalearthdata.com/downloads/](https://www.naturalearthdata.com/downloads/); [https://www.naturalearthdata.com/about/terms-of-use/] (https://www.naturalearthdata.com/about/terms-of-use/).

### Haplotype distribution and spatial-temporal genetic structure

The COX1 haplotype distribution was highly uneven, with a few widespread haplotypes and numerous low-frequency variants (Table 2 and Fig 2a). H1 was the dominant haplotype, followed by H5 and H9, and these common haplotypes were detected across multiple departments, eco-regions, collection years, and sequence sources. In contrast, most remaining haplotypes were rare and geographically restricted, occurring in one or a few localities.

**Fig 2:**
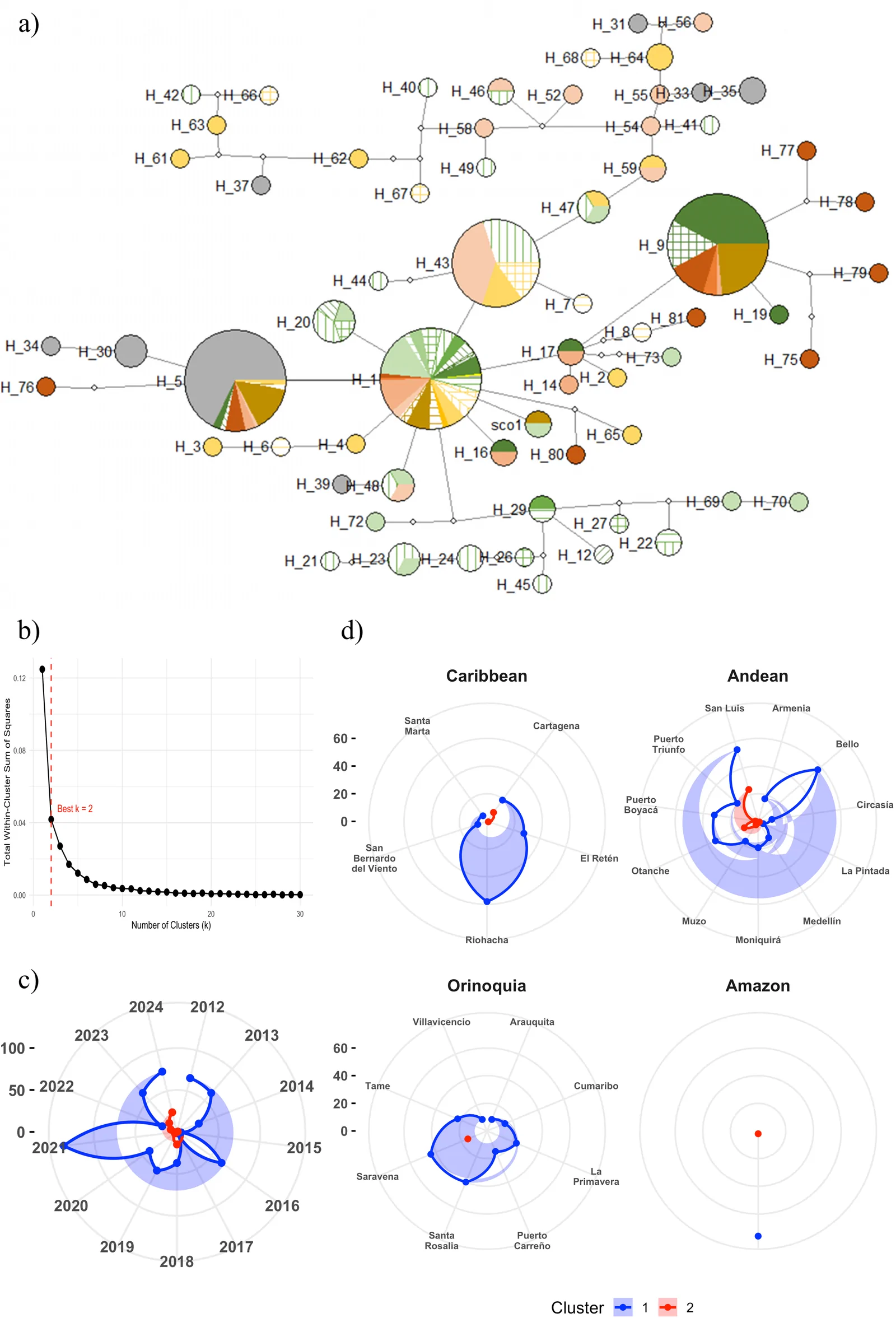
Median-joining haplotype network, K2P-based clustering, and spatial-temporal distribution of mitochondrial COX1 genetic clusters in Colombian *Ae. aegypti.* (a) Median-joining haplotype network based on COX1 sequences. Node size is proportional to haplotype frequency, and white nodes represent median vectors. Colors and fill patterns indicate collection origin according to Table 2. Colors identify departments within eco-regions, and fill patterns distinguish localities within departments: Amazon = grey (Amazonas: Leticia, solid); Andean = green shades [Antioquia, dark green: Bello (solid), La Pintada (backward diagonal), Medellín (squares), Puerto Triunfo (forward diagonal); Boyacá, medium green: Moniquirá (solid), Muzo (horizontal), Otanche (vertical), Puerto Boyacá (squares); Quindío, light green: Armenia (solid), Circasía (horizontal); Tolima: San Luis (solid)]; Caribbean = orange shades [Bolívar: Cartagena (solid); Córdoba: San Bernardo del Viento (solid); La Guajira: Riohacha (solid); Magdalena: El Retén (solid), Santa Marta (squares)]; and Orinoco = yellow shades [Arauca: Arauquita (squares), Saravena (solid), Tame (horizontal); Meta: Villavicencio (solid); Vichada: Cumaribo (vertical), La Primavera (horizontal), Puerto Carreño (squares), Santa Rosalía (solid)]. (b) Elbow analysis supporting two main K2P-based genetic clusters. (c) Temporal distribution of cluster assignments by collection year. (d) Spatial distribution of cluster assignments by locality within each eco-region. In panels (c) and (d), blue represents Cluster 1, the dominant mitochondrial component, and red represents Cluster 2, a less frequent component with a more restricted spatial-temporal distribution.

K2P-based clustering supported two main mitochondrial genetic clusters among Colombian *Ae. aegypti* COX1 sequences (Fig 2b). Cluster 1 was the dominant component and was broadly distributed across collection years, eco-regions, and most sampled localities (Fig 2c, 2d). In contrast, Cluster 2 was less frequent and showed a more restricted distribution, occurring intermittently across years and mainly in a subset of Andean, Caribbean, and Orinoco localities. Overall, this pattern indicates a widespread dominant mitochondrial background accompanied by a less common, spatially and temporally heterogeneous genetic component.

K2P-based NMDS ordination showed broad overlap among *Ae. aegypti* COX1 sequences across Colombian departments and collection years, with most individuals clustering near the center of the ordination space, indicating weak geographic and temporal structuring (Fig 3a, 3b). Hierarchical AMOVA showed that genetic variance was mainly partitioned between the two K2P-derived clusters (σ^2^ = 0.007, p < 0.001), while differentiation among departments within clusters was not supported (σ^2^ = −0.003, p = 0.999); a smaller temporal component was detected among years within departments (σ^2^ = 0.001, p < 0.001). PERMANOVA indicated that spatial-temporal predictors explained only a small fraction of K2P variation, with non-significant effects of collection year (R^2^ = 0.003, p = 0.183) and latitude (R^2^ = 0.003, p = 0.113), a weak but significant effect of longitude (R^2^ = 0.009, p = 0.009), and most variation remaining residual (R^2^ = 0.977), consistent with variance partitioning (residual variation = 0.91). The Mantel test showed no isolation-by-distance pattern (r = 0.018, p = 0.75), indicating that pairwise genetic distances did not increase linearly with geographic distance. Although PERMANOVA and dbRDA differed in which predictors were individually significant, these results are not contradictory: PERMANOVA showed that spatial-temporal variables explained little of the total K2P variation, whereas dbRDA detected subtle directional effects of longitude (p = 0.001), latitude (p = 0.003), and collection year (p = 0.001) within the constrained ordination space (Fig 3c). Overall, COX1 variation was not strongly structured by department, year, or geographic distance, but reflected two broad K2P-derived clusters with a weak multidimensional spatial-temporal signal.

**Fig 3:**
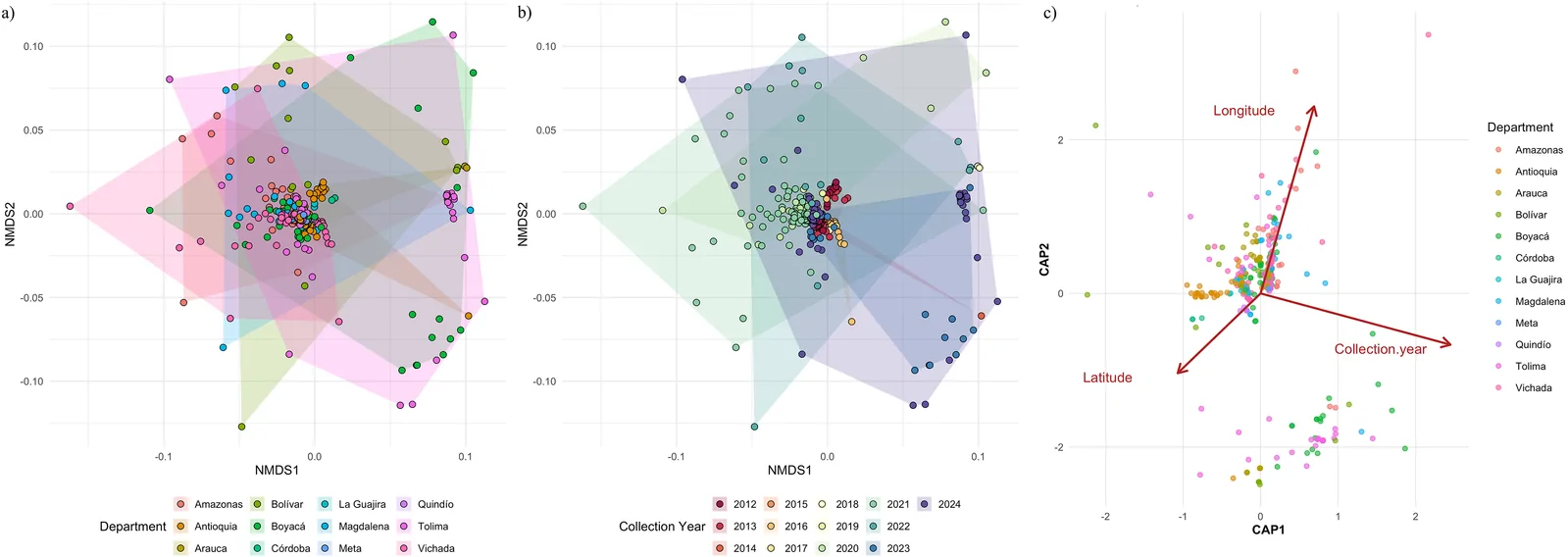
K2P-based spatial-temporal structure of Colombian *Ae. aegypti* COX1 sequences. NMDS ordination of pairwise Kimura 2-parameter genetic distances grouped by (a) department and (b) collection year. Points represent individual sequences, and shaded polygons indicate group dispersion. (c) Distance-based redundancy analysis (dbRDA) showing the association of K2P genetic distances with longitude, latitude, and collection year.

### Demographic history and ecological associations of inferred effective population size

The Bayesian skyline analysis indicated temporal variation in mitochondrial effective population size rather than continuous demographic expansion (S3 Fig). The mean trajectory showed an earlier increase, reaching its highest inferred value approximately 30 years before present, followed by a decline and relative stabilization during more recent periods. Effective population size decreased again toward the present. However, the broad 95% highest posterior density intervals, particularly for older periods, indicate substantial uncertainty in the magnitude of these changes.

Associations between BSP-inferred effective population size (Ne) and climatic variables were generally weak at the country scale (S4 Fig). Mean per-population Ne was not significantly associated with mean annual precipitation, minimum temperature, or maximum temperature, and no significant relationships were detected when interpolated monthly Ne estimates were compared with monthly precipitation, minimum temperature, or maximum temperature.

Ecoregion-stratified analyses showed that interpolated Ne was not significantly associated with monthly precipitation in the Andean, Caribbean, or Orinoco regions (Fig 4a). In contrast, monthly minimum temperature showed significant positive associations with Ne in the Andean (R = 0.25, p = 0.024), Caribbean (R = 0.64, p = 0.00075), and Orinoco regions (R = 0.35, p = 0.037), indicating higher inferred Ne under warmer minimum-temperature conditions (Fig 4b). Monthly maximum temperature showed weak positive trends in the Andean, Caribbean, and Orinoco regions, but none of these associations was statistically significant (Fig 4c). The Amazon region was not interpreted because of limited informative variation in the data. Biome-stratified analyses suggested heterogeneous recent Ne trajectories across ecological settings (Fig 4d). Interpolated Ne declined toward the present in the Andean Mountain and Wetland biomes, whereas the Humid Tropical Rainforest Biome showed an apparent increase. However, these biome-specific patterns should be considered exploratory because several categories were represented by few temporal observations, and the Seasonal Tropical and Transitional Tropical biomes did not contain enough points to infer temporal trends.

**Fig 4:**
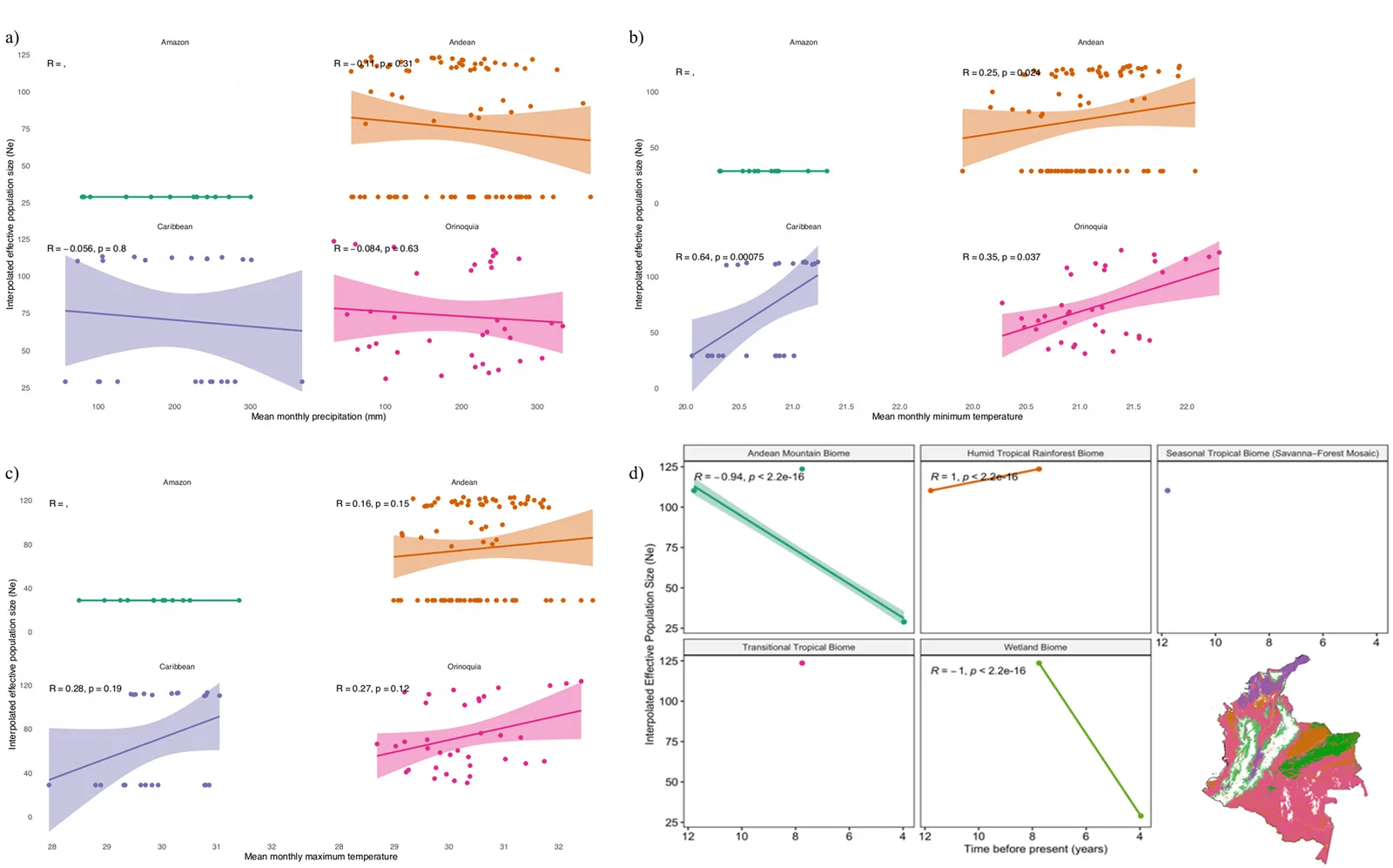
Regional climatic and biome-stratified associations with BSP-inferred effective population size (Ne) in Colombian *Ae. aegypti.* Spearman correlations between interpolated Ne and monthly (a) precipitation, (b) minimum temperature, and (c) maximum temperature across Colombian ecoregions. (d) Biome-stratified temporal trajectories of interpolated Ne based on IDEAM/IAvH biome categories (2024); biome colors are shown in the map inset. Climatic variables were extracted from WorldClim layers for 2000–2024 at 2.5 arc-min resolution (~21 km^2^).

### Anthropogenic landscape association with genetic structure

Human population density showed a detectable association with *Ae. aegypti* COX1 genetic structure, although the strength of this effect varied across sampling years (Fig 5). Year-specific dbRDA models showed that human population density explained a modest but recurrent fraction of K2P genetic variation, with significant associations in most sampling years and R^2^ values ranging from 0.032 to 0.117 among significant models (Fig 5a). The strongest effects were observed in 2012 and 2018 (R^2^ = 0.117), followed by 2019 (R^2^ = 0.102), whereas 2014 and 2020 were non-significant. The global dbRDA ordination further supported this pattern, showing a gradient among population-density classes along the constrained CAP1 axis, particularly between sparse and highly urban settings, while dispersion along the unconstrained MDS1 axis indicated additional residual genetic variation not explained by human population density (Fig 5b). Similarly, the built-up index analysis showed year-dependent structuring, suggesting that urbanization-related variables may contribute to spatial genetic heterogeneity in Colombian *Ae. aegypti* (Fig 5c). Overall, these results indicate that anthropogenic landscape features, particularly human population density and built-up environments, are associated with COX1 genetic structure, but that their influence is temporally heterogeneous rather than constant across all sampling years.

**Fig 5:**
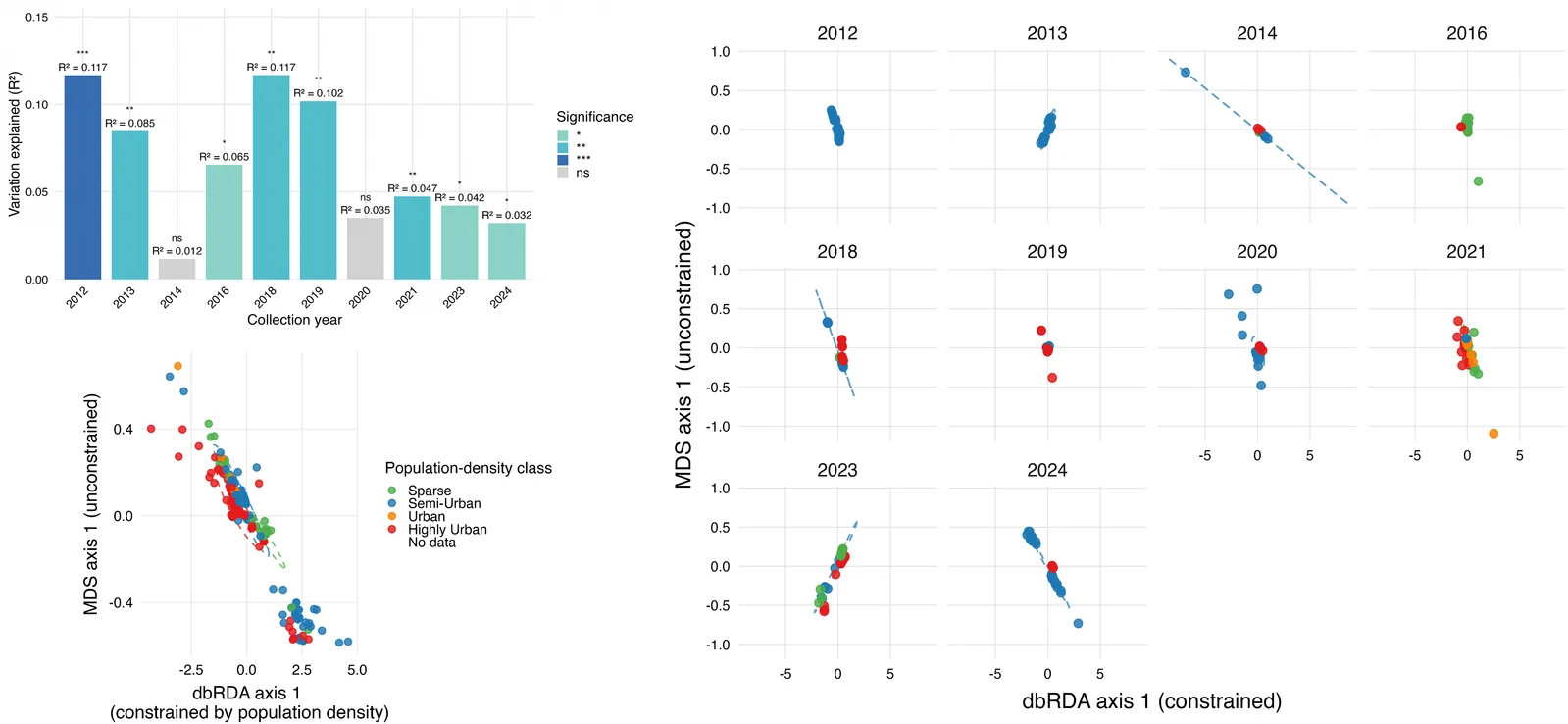
Association between anthropogenic landscape variables and K2P genetic structure in Colombian *Ae. aegypti.* (a) Year-specific dbRDA R^2^ values showing the proportion of K2P genetic variation explained by human population density; asterisks indicate statistical significance. (b) Global dbRDA ordination of genetic structure constrained by human population density, with samples colored by population-density class. (c) Year-stratified dbRDA ordination of genetic structure constrained by built-up index derived from GHSL layers. Population-density classes for panels (b) and (c) are indicated in the inset legend. Overall, the analyses indicate temporally heterogeneous associations between anthropogenic landscape features and COX1 genetic structure.

## Discussion

This study provides a country-scale mitochondrial assessment of *Aedes aegypti* in Colombia, linking COX1 diversity with spatial-temporal structure, demographic history, urbanization, climate-associated variation, and epidemiological context. The main result is that Colombian COX1 diversity is organized into two broad mitochondrial genetic clusters, rather than into clearly separated groups by locality, region, or collection year. This agrees with studies from both the native and introduced ranges of *Ae. aegypti*, including Colombia, where major mitochondrial lineages have been reported repeatedly [21,61–63]. Human population density and built-up intensity partially explained mitochondrial genetic structure, but this association changed among years. Demographic history analyses also suggested temporal variation in mitochondrial effective population size, rather than continuous demographic expansion. Together, these results indicate that Colombian *Ae. aegypti* mitochondrial diversity reflects the persistence and spatial mixing of distinct COX1 haplotype groups, with patterns consistent with human-mediated dispersal and associations with urbanization, climatic heterogeneity, and local demographic variation.

These findings fit within the broader view of *Ae. aegypti* as an invasive mosquito strongly shaped by human movement and urbanization. Genome-scale studies support an African origin for the human-specialist invasive lineage, followed by expansion through the Americas and later global dispersal associated with human movement and urban environments [2,3]. Genome-wide SNP and whole-genome datasets have also shown strong utility for reconstructing population structure, invasion routes, gene flow, and demographic history in this species [19]. Within this global framework, Colombian populations remain an important but still incompletely characterized component of the species’ invasion history.

Previous mitochondrial studies in Colombia have shown that *Ae. aegypti* genetic variation is not homogeneous across localities. Jaimes-Dueñez et al. (2015) reported two main African-related mitochondrial lineages, including a widespread lineage associated with West African ancestry and a less frequent lineage related to East African ancestry. Other local and regional studies using mitochondrial markers have detected haplotype variation or molecular differentiation among Colombian mosquito populations, particularly in northern and Andean regions [26,28,30,31,33]. However, microsatellite-based analyses suggested substantial gene flow among geographically distant Colombian cities and supported a single African origin for sampled Colombian populations [22]. This contrast is expected because mtDNA, as a maternally inherited and non-recombining marker, can preserve historical maternal-lineage signals, whereas microsatellites and genome-wide SNPs are better suited to detecting contemporary admixture, fine-scale structure, and recent gene flow [5,64,65].

The two broad COX1 clusters detected here are compatible with previous reports of mitochondrial heterogeneity in Colombia, but they should not be interpreted as definitive evidence of strong contemporary population subdivision without nuclear genomic support. The K2P and NMDS analyses showed that sequences did not form discrete groups by locality, department, or collection year, but instead clustered into two mitochondrial groups with partial geographic overlap. This pattern suggests persistence of broad maternal lineages together with spatial mixing, likely promoted by human-mediated dispersal, transport networks, trade, and recurrent movement among domestic and peri-domestic habitats. From an epidemiological perspective, these clusters may represent maternal lineages with different histories of introduction, persistence, or regional movement, potentially useful for identifying source areas, recolonization routes, or population turnover after control interventions. However, they should not be assumed to differ in arbovirus transmission potential. In Bello, Colombia, two *Ae. aegypti* lineages were affected similarly by DENV-2 infection, indicating that mitochondrial lineage differentiation does not necessarily imply differences in vector competence [66]. Testing the epidemiological relevance of the clusters detected here will require integrating mitochondrial and nuclear genomic data with vector-competence assays, insecticide-resistance profiles, viral detection, and longitudinal dengue surveillance.

Urbanization appears to contribute to mitochondrial genetic structure, but only as a partial and temporally variable driver. Human population density and built-up intensity were associated with COX1 genetic distances in 2016, 2018, 2019, 2021, and 2023, whereas other years showed weak or non-significant patterns. This suggests that the relationship between urbanization and mosquito genetic structure is not static, but is likely modulated by regional differences in human mobility, settlement connectivity, vector-control pressure, founder effects, and local demographic turnover [3]. Eco-regional demographic analyses also showed heterogeneous trajectories of mitochondrial effective population size, and Spearman correlation results indicated that these temporal patterns were not uniform across ecological regions. Thus, urbanization-associated genetic structure should be understood as part of a broader ecological and regional demographic process rather than as an isolated effect.

This interpretation is especially relevant in Colombia, where demographic change and urbanization are strongly regionally structured. The Departamento Administrativo Nacional de Estadística (DANE) demographic-change estimates, based on the 2018 National Population and Housing Census, show spatially heterogeneous population dynamics across the country, with projections of population growth and demographic components at national and regional scales for 2018–2050 (Departamento Administrativo Nacional de Estadística (DANE), 2025; https://www.dane.gov.co/index.php/estadisticas-por-tema/demografia-y-poblacion/estimaciones-del-cambio-demografico). The Andean region concentrates a large proportion of Colombia’s population and major urban systems, whereas the Amazon, Orinoco, Pacific, and parts of the Caribbean show different trajectories related to lower settlement density, dispersed populations, migration dynamics, or localized urban growth. Accordingly, human population density in our analyses likely reflects not only local urbanization intensity, but also broader differences in demographic concentration, settlement history, and connectivity.

Colombian *Ae. aegypti* COX1 diversity was primarily partitioned between two broadly distributed mitochondrial clusters, with limited differentiation by locality, department, collection year, or geographic distance. Human population density and built-up intensity explained a small component of this mitochondrial variation, although the strength of these associations differed among sampling periods. This pattern is consistent with the close ecological relationship between *Ae. aegypti* and human settlements, where artificial water containers, dense housing, and other built environments can support local persistence, while transport networks and human mobility may facilitate dispersal among urban areas [2,67]. Nevertheless, these associations are correlative, and the remaining genetic variation may also reflect drift, localized bottlenecks, recurrent introductions, demographic changes, and heterogeneous vector-control pressures.

The demographic history analyses provide a complementary temporal perspective. Rather than supporting continuous demographic expansion, they suggest variation in mitochondrial effective population size, with an older increase followed by reduction and relative stabilization toward recent generations. This pattern may reflect historical establishment in human-modified landscapes followed by demographic constraints, local bottlenecks, vector-control pressure, or extinction–recolonization dynamics. Because these estimates are derived from COX1 and depend on the selected mitochondrial substitution rate, they should be interpreted as approximate, calibration-dependent reconstructions of maternal-lineage history rather than direct estimates of census population size, vector density, or genome-wide effective population size. Together with the spatial analyses, these results suggest that demographic history, urbanization, and eco-regional heterogeneity have jointly shaped Colombian *Ae. aegypti* populations.

The association between mitochondrial demographic variation and minimum temperature should be interpreted cautiously. Although country-scale associations were non-significant, the positive regional correlations suggest that minimum temperature may be associated with mitochondrial demographic variation under specific ecological conditions. Nevertheless, these associations were exploratory and do not establish a causal relationship. Minimum temperature remains biologically relevant because warmer lower-bound temperatures may reduce thermal constraints on mosquito survival, development, and adult activity and may influence arbovirus extrinsic incubation dynamics [68]. This may be particularly important in Colombia, where elevation strongly structures thermal conditions and cooler nights can limit vector persistence in some areas. Climate-change evidence for Latin America, including Colombia, indicates increased heat exposure, altered precipitation regimes, and growing health risks associated with climate variability and extreme events [69]. Colombian studies have shown that arbovirus transmission is influenced by local temperature, microclimate, entomological conditions, and ecological heterogeneity [70,71]. In the present study, demographic variation refers specifically to temporal changes in BSP-inferred mitochondrial effective population size. Although minimum temperature is biologically relevant to *Ae. aegypti* ecology [71], its associations with inferred Ne were non-significant at the country scale, whereas significant positive correlations were detected in monthly analyses of the Andean, Caribbean, and Orinoco regions. Nevertheless, these findings do not establish that warmer temperatures directly contributed to the inferred demographic history. These relationships should instead be regarded as exploratory correlations, without evidence of direct climatic or climate-change causality.

The dengue epidemiological context in Colombia reinforces the importance of interpreting these genetic patterns within regionally heterogeneous environments. The INS dengue bulletin for 2025 reported strong temporal and regional heterogeneity during the 2023–2025 epidemic cycle, which has registered the highest number of dengue cases in recent Colombian history. Municipal time-series analyses identified three dengue-incidence clusters: cluster 1 was mainly distributed in Amazonía, Caribe, and Orinoco, whereas clusters 2 and 3 were predominantly Andean (Instituto Nacional de Salud (INS) de Colombia, 2025). These epidemiological clusters suggest that *Ae. aegypti* populations are embedded in regionally distinct demographic, climatic, and urban contexts. In the Andean region, dense urban corridors and high human connectivity may favor persistence, recolonization, and gene flow, whereas in Amazonian, Caribbean, and Orinoco settings, outbreak dynamics may be more strongly affected by climatic seasonality, dispersed settlement patterns, local environmental suitability, and episodic introductions. The INS report also indicates that 85.4% of Colombian municipalities have conditions for DENV transmission and that *Ae. aegypti* was reported in 67.6% of municipalities by 2024, providing a broad epidemiological context for the weak geographic-temporal partitioning observed in COX1 variation (Instituto Nacional de Salud (INS) de Colombia, 2025).

Several limitations should be considered. COX1 is useful for assessing maternal lineage diversity and broad phylogeographic patterns, but it has limited resolution for contemporary gene flow, admixture, and fine-scale structure. Thus, the two mitochondrial clusters and demographic patterns detected here should not be interpreted as definitive evidence of genome-wide population subdivision. Uneven sampling across localities and years may also influence genetic distances, ordination patterns, constrained analyses, and demographic estimates. Finally, human population density and temperature should be viewed as proxies for broader ecological processes, including urbanization, connectivity, mobility, settlement history, and regional climatic heterogeneity.

Despite these limitations, this study provides a useful mitochondrial baseline for genomic surveillance of *Ae. aegypti* in Colombia. The detection of broad mitochondrial structure, weak geographic-temporal partitioning, urbanization-associated genetic variation, and temporal changes in mitochondrial effective population size suggests that Colombian populations are shaped by historical lineage retention, human-mediated dispersal, regional demographic heterogeneity, and local ecological processes. Future studies should integrate genome-wide SNPs, whole-genome sequencing, or dense microsatellite panels with standardized entomological sampling, vector-control records, insecticide-resistance monitoring, human mobility data, viral circulation, and climate anomalies such as El Niño and La Niña. Such an approach would help distinguish historical mitochondrial structure from contemporary dispersal, identify recurrent introductions or local population replacement, and test whether genetic changes are associated with dengue incidence, environmental suitability, or control pressure.

## Conclusion

This study provides a country-scale assessment of mitochondrial COX1 diversity in *Aedes aegypti* from Colombia. Genetic variation was primarily partitioned between two broadly distributed K2P-derived mitochondrial clusters, with little support for differentiation among departments and only a small temporal component. These results indicate the coexistence and geographic mixing of distinct maternally inherited haplotype groups but should not be interpreted as evidence of contemporary genome-wide gene flow. Human population density and built-up intensity explained a small component of mitochondrial variation, and the strength of these associations differed among sampling periods, suggesting that urban heterogeneity may influence mitochondrial spatial patterns without establishing a direct causal relationship. The Bayesian skyline analysis suggested an earlier increase in mitochondrial effective population size followed by decline and relative stabilization, although broad uncertainty intervals and substitution-rate dependence limit precise interpretation of its magnitude and timing. Climatic associations were weak, directionally inconsistent, and non-significant, providing no evidence that temperature contributed directly to the inferred demographic trajectory. Overall, these findings provide a country-scale mitochondrial baseline for future genome-wide, entomological, and epidemiological studies of *Ae. aegypti* dispersal and population dynamics in Colombia.

## Supporting information

S1 Table Complete metadata for the 717 *Aedes aegypti* COX1 records included in this study, including sample code, GenBank accession number, department, locality, geographic coordinates, collection year, original code, and source. For shared haplotypes represented across multiple locality-year combinations, separate metadata rows were retained for each sampling event.
(XLSX)

S1 Data R script used to retrieve and curate GenBank metadata for Colombian *Aedes aegypti* COX1 sequences using the NCBI Entrez utilities through the *rentrez* package, including accession retrieval, batch downloading of GenBank and FASTA records, metadata extraction, incorporation of curated locality information from S1 Table, and expansion of shared haplotypes across multiple sampling events. (R)

S2 Data R workflow used during iterative curation of the compiled *Aedes aegypti* COX1 dataset, including removal of gap characters prior to BLASTN validation against a mitochondrial COX1 reference, retention of BLAST-supported query regions, MAFFT alignment, evaluation of alternative gap thresholds for trimming poorly aligned terminal regions, and final filtering of alignment positions with gaps in ≥45% of sequences. (R)

S3 Data R script used to verify coding-frame integrity of the final *Aedes aegypti* COX1 alignment, including removal of non-ACGT characters, translation under the invertebrate mitochondrial genetic code, detection and consensus-based repair of internal stop codons, and export of the repaired nucleotide alignment. (R)

S4 Data Ecological and climatic associations with BSP-inferred effective population size in Colombian *Aedes aegypti.* This R script evaluates exploratory associations between Bayesian Skyline Plot-derived effective population size (Ne) and ecological-climatic variables across Colombia. The workflow includes processing BSP skyline estimates, interpolating Ne through time, extracting WorldClim precipitation, minimum temperature, and maximum temperature values for 2000–2024 at 2.5 arc-min resolution, and testing annual, monthly, and ecoregion-stratified Spearman correlations. The script also assigns sampling localities to IDEAM/IAvH biome categories (2024), evaluates temporal trends in Ne within ecological units, and exports summary tables and plots used to generate the Fig 4 and S4 Fig.
(R)

S5 Data Anthropogenic landscape associations with K2P genetic structure in Colombian *Ae. aegypti.* This R script evaluates the association between COX1 K2P genetic distances and anthropogenic landscape variables derived from GHSL/GHS datasets. The workflow includes extraction of human population density and built-up index values for sampling localities, assignment of population-density classes, year-specific dbRDA models, global dbRDA constrained by human population density, year-stratified dbRDA constrained by built-up index, and export of summary tables and plots used to generate Fig 5.
(R)

S1 Fig Geographic and temporal distribution of Colombian *Aedes aegypti* COX1 sequences included in this study. (a) Map of Colombia showing sampled localities, with bubble size proportional to the number of COX1 sequences per locality. The inset indicates the geographic position of Colombia in South America. (b) Distribution of sequence counts by department, locality, and collection year. Collection years are represented by the color gradient. This figure complements the sampling information summarized in Tables 1 and S1. Administrative boundaries and country outlines were obtained from Natural Earth via the `rnaturalearth` and `rnaturalearthhires` R packages. Natural Earth data are in the public domain. Data and terms: [https://www.naturalearthdata.com/downloads/](https://www.naturalearthdata.com/downloads/); [https://www.naturalearthdata.com/about/terms-of-use/](https://www.naturalearthdata.com/about/terms-of-use/).
(TIF)

S2 Fig Summary of the gap-threshold filtering procedure applied to the compiled Aedes aegypti COX1 alignment. **(a)** The trade-off curve shows the effect of alternative gap thresholds on retained alignment positions, retained sequences, and mean ungapped sequence length. **(b)** The critical-gap profile shows the distribution of highly gapped positions across the alignment, indicating that most problematic sites were concentrated at the alignment ends, with fewer isolated internal positions. **(c)** The histogram summarizes the distribution of ungapped sequence lengths.
(TIF)

S3 Fig Bayesian skyline reconstruction of mitochondrial demographic history in Colombian *Aedes aegypti.* The dashed line represents the mean inferred effective population size through time, and the shaded area represents the 95% highest posterior density interval. Time is expressed as years before present. Estimates represent mitochondrial maternal-lineage history and should not be interpreted as census population size or genome-wide effective population size.
(TIF)

S4 Fig Country-scale associations between BSP-inferred effective population size (Ne) and climatic variables in Colombia. **(a)** Annual and monthly associations between Ne and precipitation. **(b)** Annual and monthly associations between Ne and minimum temperature. **(c)** Annual and monthly associations between mean per-population Ne and maximum temperature. Upper panels show relationships between Ne and mean annual climatic variables, whereas lower panels show relationships between interpolated monthly Ne and monthly climatic variables. Shaded areas represent 95% confidence intervals around fitted linear trends.
(TIF)
